# Castration, Etc.

**Published:** 1853-08

**Authors:** A. H. Van Norstrand

**Affiliations:** Jefferson


ART II.—*Castration performed with the Thumb Nail.*—By A. H. VAN NORSTRAND, M. D.

I wish to call to your notice, as well as the rest of the profession, a new Surgeon, a new operation, and a new instrument.

The Surgeon by name Willcox, a turner by trade, the operation castration, and the instrument a thumb nail.

The circumstances are as follows: on the morning of March, 14th ult., about eight o'clock, A. M., my partner (Dr. Mack) and myself were called in some haste to the jail; when we arrived, we found the man above named, lying upon his bed, his garments bloody and the scrotum detesticled; the left testicle has the cord still attached, it having been pulled out from the abdominal ring; the other testicle had no cord attached, it having been torn off in his efforts to extract it; both testicles were hanging upon the cell door. I interrogated him as to the manner in which he performed the operation, and he answered as follows: "It was an awful operation, but I was compelled to do it, that I might conquer the world. I squeezed one in the front part of the bag and then took my thumb nail" (which was long and very sharp, and actually was the instrument used) "and cut down upon it till I had a hole large enough to get the stone through, I then took hold of it and pulled it out, cord and all; I then rested a while, but finally concluded that I wanted to see how the other one looked. So I cut through the partition (septum scroti) and took out the other, but I do not feel satisfied with that for I could not pull out the cord, it seemed to be rotten and tore off from the stone, still I think it will do, and as soon as I get well there will be no man that can stand before me." The hemorrhage had ceased, and the parts were somewhat swollen and above the natural temperature. I ordered ℥ij. Mur. Amon. dissolved in a quart of cold water applied to the parts by dipping clothes in the solution and placed upon the parts, and frequently changed. This was continued until the wound commenced to granulate, it was then discontinued, and further appliances were deemed useless. The wound completely healed in thirty days from the date of its production.

This man is about 50 years of age, is 6 feet 8 inches high, and possessed of great muscular power and determination of will, is insane on religious subjects generally, and spiritual rappings in particular; he has frequently requested me to furnish him another pair of testicles. He has told me that he had another pair growing, and would soon be as good as ever.

JEFFERSON, June 22, 1853.

